# Lung-to-finger circulation time can be measured stably with high reproducibility by simple breath holding method in cardiac patients

**DOI:** 10.1038/s41598-021-95192-3

**Published:** 2021-08-05

**Authors:** Tomoyuki Tobushi, Takatoshi Kasai, Masayuki Hirose, Kazuhiro Sakai, Manabu Akamatsu, Chizuru Ohsawa, Yasuko Yoshioka, Shoko Suda, Nanako Shiroshita, Ryo Nakamura, Toshiaki Kadokami, Takeshi Tohyama, Kouta Funakoshi, Kazuya Hosokawa, Shin-ichi Ando

**Affiliations:** 1 Department of Cardiovascular Medicine, Saiseikai Futsukaichi Hospital, Chikushino, Japan; 2 Department of Cardiovascular Medicine, Juntendo University Graduate School of Medicine, Tokyo, Japan; 3 Cardiovascular Respiratory Sleep Medicine, Juntendo University Graduate School of Medicine, Tokyo, Japan; 4 Center for Clinical and Translational Research, Kyushu University Hospital, Fukuoka, Japan; 5 Imaging Device Development, Fuji Xerox Co., Ltd., Kanagawa, Japan; 6 Sleep Apnea Center, Kyushu University Hospital, 3-1-1 Maidashi, Higashi-ku, Fukuoka, 812-8582 Japan; 7 Department of Cardiovascular Medicine, Kyushu University Hospital, Fukuoka, Japan

**Keywords:** Blood flow, Heart failure, Biomedical engineering

## Abstract

Lung to finger circulation time (LFCT) has been used to estimate cardiac function. We developed a new LFCT measurement device using a laser sensor at fingertip. We measured LFCT by measuring time from re-breathing after 20 s of breath hold to the nadir of the difference of transmitted red light and infrared light, which corresponds to percutaneous oxygen saturation. Fifty patients with heart failure were enrolled. The intrasubject stability of the measurement was assessed by the intraclass correlation coefficient (ICC). The ICC calculated from 44 cases was 0.85 (95% confidence interval: 0.77–0.91), which means to have “Excellent reliability.” By measuring twice, at least one clear LFCT value was obtained in 89.1% of patients and the overall measurability was 95.7%. We conducted all LFCT measurements safely. High ICCs were obtained even after dividing patients according to age, cardiac index (CI); 0.85 and 0.84 (≥ 75 or < 75 years group, respectively), 0.81 and 0.84 (N = 26, ≥ or < 2.2 L/min/M^2^). These results show that our new method to measure LFCT is highly stable and feasible for any type of heart failure patients.

## Introduction

As the number of patients with heart failure (HF) increases, there is an increasing need to manage patients with HF more efficiently. Although taking the cardiac output (CO) into consideration of the therapy process is one of the key elements of HF management, the methods to measure CO in actual clinical setting is limited due to the following reasons: catheter method^1^ include invasive procedure, MRI method^2^ is expensive, and echocardiography method^3^ is strongly dependent on the examiner's skill and patients’ physical characteristics. The noninvasive CO measurement methods currently in use including the echocardiography method and MRI method are summarized in Table 1. Methods other than echocardiography and MRI method are mainly used in intensive care units or experimental conditions. Thus, a new method which can be used at home or in a general ward is needed.

**Table 1:** Non-invasive methods for CO measurement.

Non-invasive CO measurement methods
Echocardiogram^4^
Cardiac MRI^2^
Volume clamp method^5^
Impedance cardiography^6^
Bioreactance method^7^
Pulse wave transit time analysis^8^
Inert gas rebreathing technique^9^

CO, cardiac output; MRI, magnetic resonance imaging.

Previous studies showed that lung to finger circulation time (LFCT) could be calculated using simple devices correlated with CO^10–12^. We also reported that LFCT calculated using night time polygraphy (respiratory movement and blood oxygen level) could be an indicator of CO^13,14^, and the LFCT with this method could reflect the change of CO during the process of treatment in patients with HF with reduced ejection fraction^15^.

On the other hand, the method that other researchers and we used principally depended on night-time measurement of breathing, and the application was limited only for the patients who undergo nocturnal polygraphy and, therefore, was not suitable for daytime measurement in the daily clinical practice. Thus, we started to develop a new device to measure LFCT by 20-s breath hold. We previously investigated the relationship between cardiac index (CI) and 1/LFCT. And we found that there was a positive tendency between CI and 1/LFCT in patients whose finger temperature > 31 °C^16^. Combined with previous studies^10–12^ we consider that LFCT reflects cardiac output. For advance further study using our method, we definitely need to verify the reproducibility, and find the minimum number of repetitions of the procedures within the same patient.

Accordingly, the purposes of this study are to verify the stability of LFCT measurement using our method by accessing the intrasubject reproducibility and to clarify the necessary minimum number of LFCT measurements for proper assessment of the LFCT value.

## Methods

The current study was a prospective double center observational and non-randomized study conducted in Saiseikai Futsukaichi hospital and Juntendo University hospital after obtaining approval from each ethics review board and written informed consent from all patients. This study was registered in University Hospital Medical Information Network Clinical Trials Registry (UMIN-CTR) in Japan (UMIN000037908). All methods in this study were performed in accordance with the relevant guidelines and regulations.

### Patients enrollment

We enrolled HF patients over 20 years old who were on the treatment and with NYHA Class I-III symptom among in-hospital and out-hospital patients. We excluded patients as below: (1) In the acute phase of cardiovascular disease (< 7 days from the onset), (2) On oxygen inhalation, (3) Measurement device could not be equipped on the second and third finger of the right hand, (4) Patients with impaired recognition, (5) Patients with a permanent pacemaker, (6) Patients on hemodialysis, (7) When the investigator judged as ineligible for this study.

### Target sample size

As these studies were exploratory investigations, we set the target sample size from the viewpoint of feasibility. The number of in-patients and out-patients with HF had been estimated at 100 patients/year and 200 patients/year in each hospital, and the research period had been determined as for 4 months. This means that we could expect to encounter 200 HF patients at most. However, since the HF patients are usually elderly, and a previous review had indicated that up to 79% of HF patients were frail^17^, we predicted that many of them would not be able to stop breathing for as long as 20 s repeatedly. And many patients were expected to be inappropriate for repeated breath hold because they would have such diseases as severe hypertension, severe aortic valve stenosis, pre-operative status, or severe HF. Considering these factors, we estimated the rate of enrollable patients would be about 20–30% of total HF patients. Thus, we considered that we would be able to enroll 20 to 30 patients in each hospital and eventually set the target sample size as 50 in total.

### The LFCT measurement device

**Figure 1:**
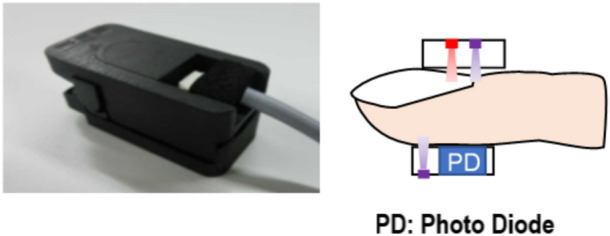
The image of the device. We set this device to a fingertip and measured LFCT. LFCT: lung to finger circulation time, PD: photo diode.

We used the device and method of measurement of LFCT as in our previous study^16^. In brief, the device equipped with a vertical-cavity surface-emitting laser sensor (Fuji Xerox Co., Kanagawa, Japan) was positioned at a fingertip. The sensor was composed of two wavelengths of laser emitting parts and photodiode parts (Fig. 1). This device utilized a two-wavelength transmitted light (red and infrared light) and a one-wavelength reflected light (infrared light). From the transmitted light, this device calculates the difference of photodiode signal levels between the direct current component of the transmitted red light and infrared light, which corresponds to percutaneous oxygen saturation (S_p_O_2_).

### Procedure for LFCT measurement and data exclusion criteria

**Figure 2:**
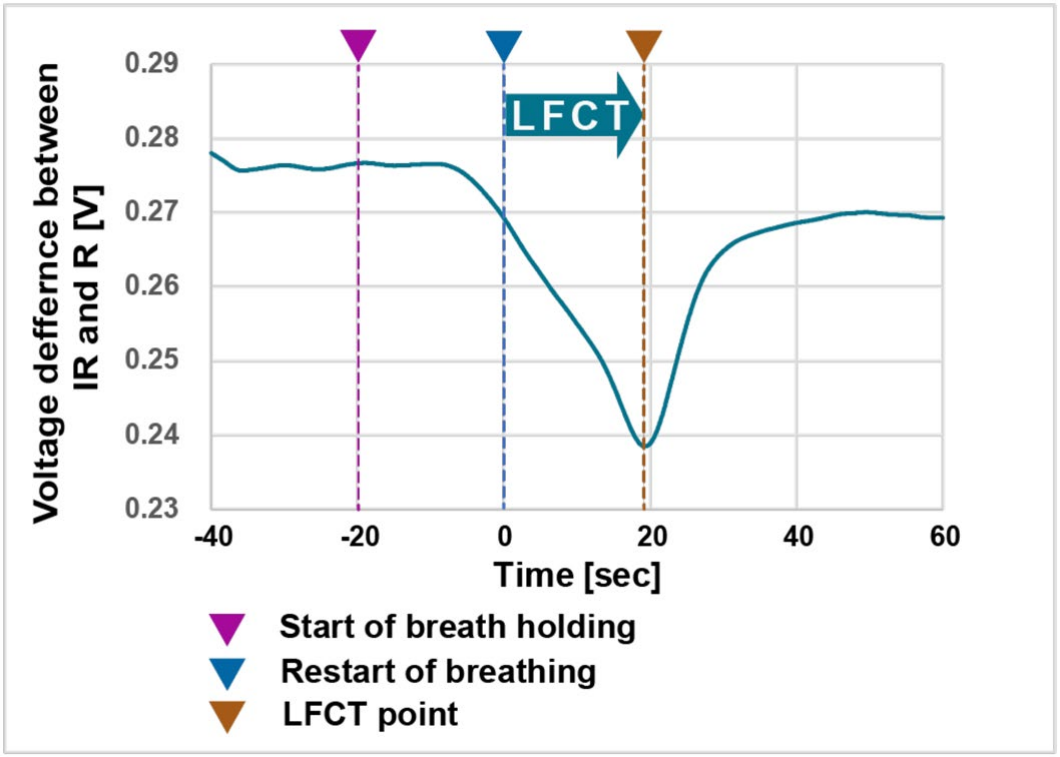
Typical trace during LFCT measurement. We asked the patients to hold their breath for 20 s. After breath holding, they restart their breathing according to the sign from the attending researcher. The time from the restart of breathing to the minimum value of the difference of R and IR (≈SpO_2_) was defined as LFCT. In this case, LFCT was 19.2 s. LFCT: lung to finger circulation time, R: red light, IR: infrared light.

Patients were asked to lay down, and an air-flow sensor (TR-101A, Nihon Kohden Corp., Tokyo, Japan) was set on the nose. We measured fingertip temperature using a portable non-contact thermometer (PT-3S, OPTEX FA CO., LTD, Kyoto, Japan). As we had found the inverse of LFCT correlated with CO when the fingertip temperature was more than 31 °C in our previous study^16^, we set the threshold of finger temperature at 32 °C. If the temperature was below 32 °C, we warmed the wrist and finger using an electrically heated glove, and we started to measure LFCT after the fingertip temperature became over 32 °C. After the signal from the fingertip device settled, patients were asked to hold breath for 20 s after expiration. After 20 s, the patients restarted their spontaneous breathing, and we measured the LFCT as the time between the restarting point to the nadir of the difference of photodiode signal levels between the direct current component of the transmitted red light and infrared light. After enough resting time, we repeated the measurement of LFCT four times in total. In case that the patient could not repeat holding breath properly anymore, we finished the measurement at the point. A representative LFCT curve is shown in Fig. 2. We recorded all respiratory flows and LFCT curve data and judged whether the data was appropriate or not for the off line analysis. We excluded the data if no clear singular nadir with the minimum value appeared in the LFCT curve, and if the nadir value occurred within 5 s from restarting of breath. All LFCT curves were reviewed and judged LFCT value by 2 cardiologists (TT, KH), who were blinded to all patients' information.

### Echocardiography and N-terminal pro-brain natriuretic peptide

We obtained left ventricular ejection fraction, end diastolic volume and end systolic volume data from echocardiography data conducted within 4 months. Left ventricular end diastolic volume, end systolic volume, and ejection fraction was determined by Teichholdz method^18^ or Simpson’s biplane method according to the recommendations of the American Society of Echocardiography^19^. Cardiac outputs were estimated by echocardiography using the pulse wave Doppler^4^. We measured velocity time integral (VTI) using an apical 5-chamber view or apical 3-chamber view with the pulsed wave doppler placed at the left ventricular outflow tract. We calculated cross sectional area at the same place and calculated stroke volume as below:

$$\text{Stroke volume= Cross sectional area (cm}^{2}) \times \text{ VTI (cm)}$$

Cardiac output was calculated as below:

$$\text{Cardiac output= Stroke volume (ml) } \times \text{ heart rate (bpm)}$$

Cardiac index was also calculated, by correcting CO for body surface area.

We also obtained the data of plasma N-terminal pro-brain natriuretic peptide (NT-proBNP).

### Statistical analysis

The primary evaluation item was LFCT reproducibility within the same patient and the secondary evaluation item was the probability of measurement of LFCT. Primary evaluation item was judged by calculation of the intraclass correlation coefficient which represents LFCT reproducibility within one patient. By measuring the LFCT values repeatedly, the intraclass correlation coefficient (ICC)^20–22^, the 95% confidence intervals on both sides of the ICC, and the standard error of the measurement (SEM) were calculated using one-way analysis of variance (ANOVA) model assuming subjects as random effects to evaluate the reliability of the measurement. In order to calculate the ICC when the number of measurements differs in every subject, we referred to the method^20^ and calculated the ICC using the PROC MIXED program in SAS9.4 (SAS Institute Inc., Cary, NC, USA). We further analyzed ICC in the subgroup classified according to age, cardiac index by echocardiography. Since, 75 years old is one indicator for the elderly in Japan, we used 75 years as the cut-off value for the age subgroup^23^. We divided cardiac function by cardiac index 2.2 L/min/m^2^ measured by echocardiogram, which is a widely used value for Forrester classification.

We measured the repeated times of measurements by we could first attain the clear peaky LFCT value.

The analyses were performed using SAS9.4 (SAS Institute Inc., Cary, NC, USA). Data are shown as mean ± standard deviation.

## Results

### Patients background

**Figure 3:**
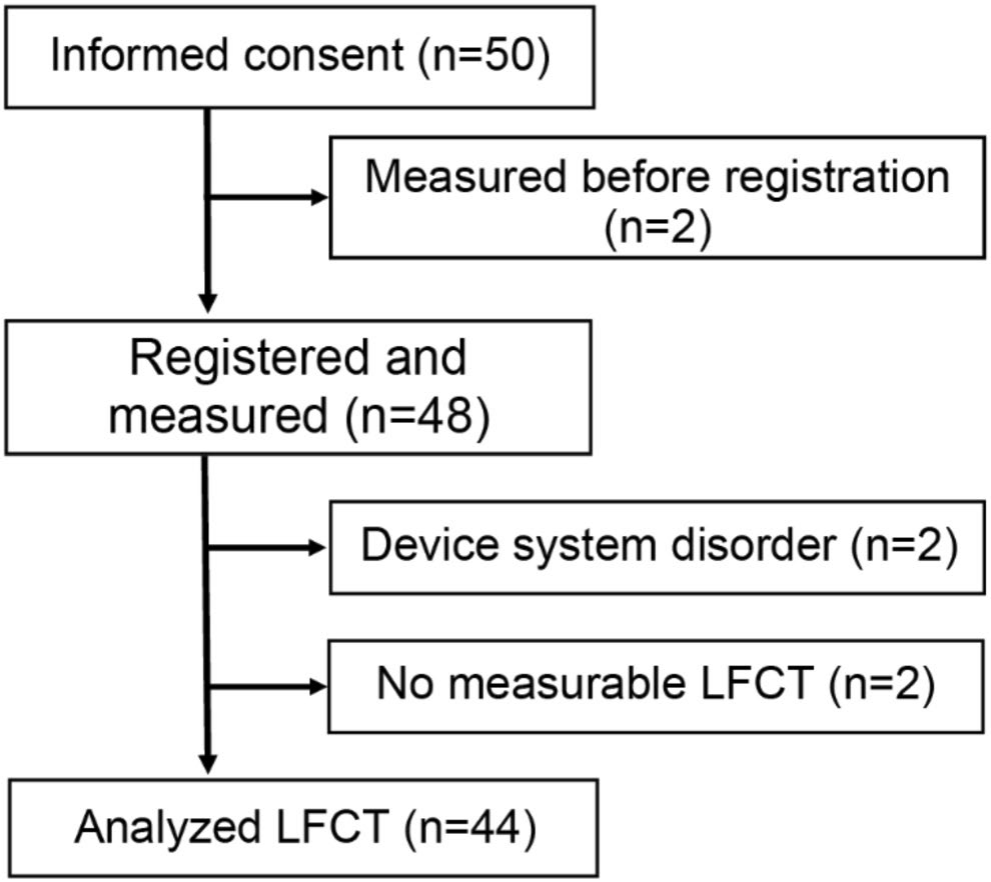
Patients flow diagram. We obtained informed consent from 50 patients. Two patients were excluded because they were accidentally measured LFCT before system registration. After registration, 48 patients were measured LFCT. Two patients were excluded because of the device system disorder. Finally, we evaluated LFCT from 46 patients; among them, 2 patients have no measurable LFCT. LFCT: lung to finger circulation time.

We enrolled 50 patients (20 patients in Juntendo University Hospital and 30 patients in Saiseikai Futsukaichi hospital) (Fig. 3). We excluded 2 cases in which case LFCT were accidentally measured before written informed consent was obtained and analyzed the remaining 48 cases. The mean age was 69.6 ± 14.1 years old and 64.6% were men. Three patients were in NYHA Class I, 42 patients in NYHA Class II, and 3 patients in NYHA Class III. NT-proBNP was 1028.5 [20–5, 110] pg/ml (median [minimum – maximum]). Other clinical backgrounds are shown in Table 2.

**Table 2:** Clinical characteristics of subjects.

Characteristics	
n	48
Men (n (%))	31 (64.6)
Age (years)	69.6 ± 14.1
Height (cm)	160.3 ± 10.1
Weight (kg)	60.8 ± 12.5
**NYHA Class (n (%))**	
I	3(6.3)
II	42(87.5)
III	3(6.3)
NTproBNP(pg/ml) median [minimum − maximum]	1028.5 [20–5, 110]
**Echocardiography**	
Ejection fraction (%) (n = 42)	55.9 ± 15.1
Estimated end diastolic volume (ml) (n = 28)	119.2 ± 49.7
Estimated end systolic volume (ml) (n = 28)	65.3 ± 36.2
Estimated cardiac index (L/min/m^2^) (n = 28)	2.4 ± 0.6

Values are mean ± SD, NYHA, New York Heart Association; NTproBNP, Nterminal pro-brain natriuretic peptide.

We excluded 2 cases from further analysis due to device system disorder. Among the remaining 46 cases, we counted the number of valid measurements in each patient. We obtained valid measurements in all 4 trials in 27 patients (58.7%), in only 3 trials in 6 patients (13.0%), in only 2 trials in 9 patients (19.6%), only once in 2 patients (4.3%), and no valid measurement in 2 patients (4.3%). Accordingly, the measurability of LFCT, which means we could measure at least one LFCT in continuous four trials, was 95.7% (44 cases in 46 cases).

### Echocardiography characteristics

Echocardiography was performed in 42 cases. The mean ejection fraction was 55.9 ± 15.1%. The left ventricular volume was assessed only in 28 cases. Mean end diastolic volume and end systolic volume was 119.2 ± 49.7 ml and 65.3 ± 36.2 ml, respectively. And calculated mean cardiac index was 2.4 ± 0.6 ml/min/m^2^.

Primary evaluation item: the intraclass correlation coefficient (ICC) as LFCT reproducibility within the same patient.

**Figure 4:**
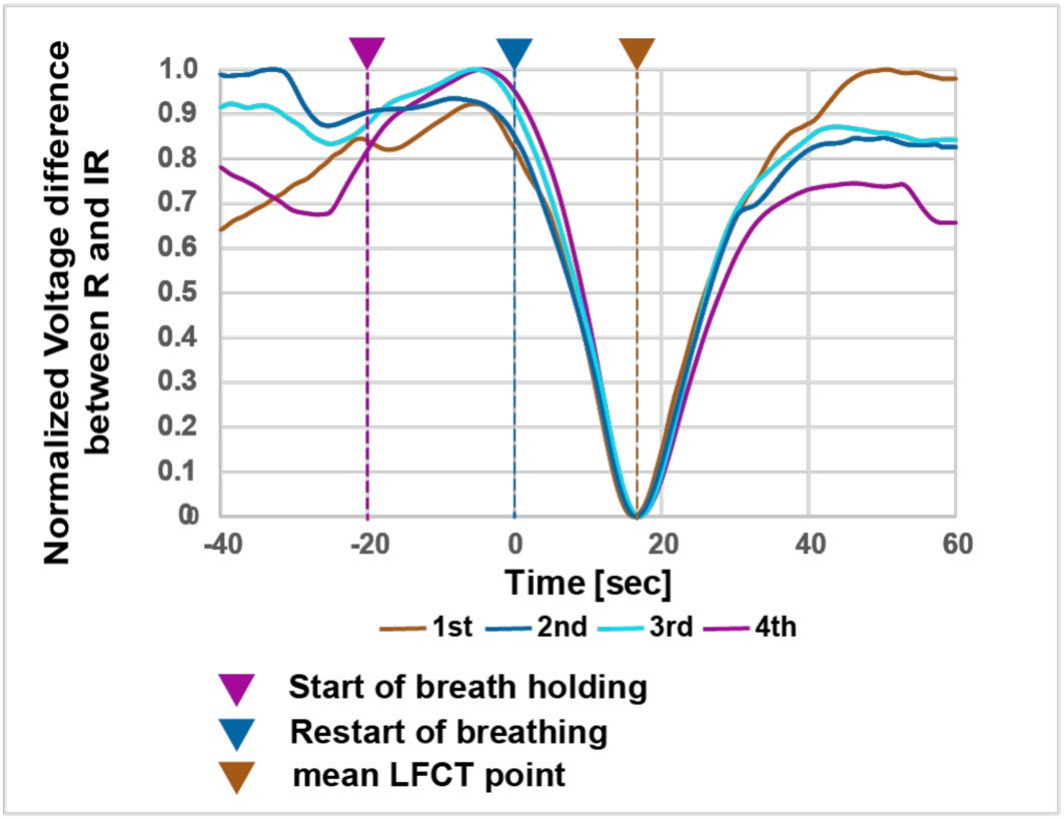
The representative patient data. Four LFCT curves were overwritten. We normalized the voltage difference to make each LFCT value clear in this figure. We calculated the maximum difference between R and IR to 1 and minimum to 0 in each session. The patient was measured LFCT for 4-times. There were clear singular nadirs in LFCT curves. Each LFCT value was 16.14, 16.50, 16.96, and 16.76 (seconds), respectively. This case showed high reproducibility of LFCT. LFCT: lung to finger circulation time, R: red light, IR: infrared light.

We showed a representative case in Fig. 4. In this case, we measured LFCT four times and the LFCT was 16.14, 16.50, 16.96, and 16.76 (seconds), respectively. In this case, LFCT is highly reproducible. The ICC calculated from 44 cases was 0.85 (95% confidence interval:0.77–0.91) and the SEM of the LFCT was 2.71.

### Subgroup analysis

We divided patients according to age (≥ or < 75 years old), cardiac index by echocardiography (N = 28: ≥ or < 2.2 L/min/M2). We calculated ICC respectively and found that ICC was not different between the groups: 0.85 and 0.84 (≥ 75 years old and < 75 years old group, respectively), 0.81 and 0.84 (≥ 2.2 L/min/M^2^ and < 2.2 L/min/M^2^, respectively) (Table 3).

**Table 3:** subgroup analysis.

Age	n	ICC	95% CI	SEM
≥ 75 years old	18	0.85	0.72–0.94	2.564
< 75 years old	26	0.84	0.73–0.92	2.8
**Cardiac index**				
≥ 2.2L/min/m^2^	17	0.81	0.65–0.92	3.232
< 2.2 L/min/m^2^	9	0.84	0.61–0.96	2.548

ICC: intraclass correlation coefficient, 95%CI: 95% Confidence Interval, SEM: standard error of the measurement, CI: cardiac index.

Secondary evaluation item: the number of the first session where a measurable LFCT value was obtained.

**Figure 5:**
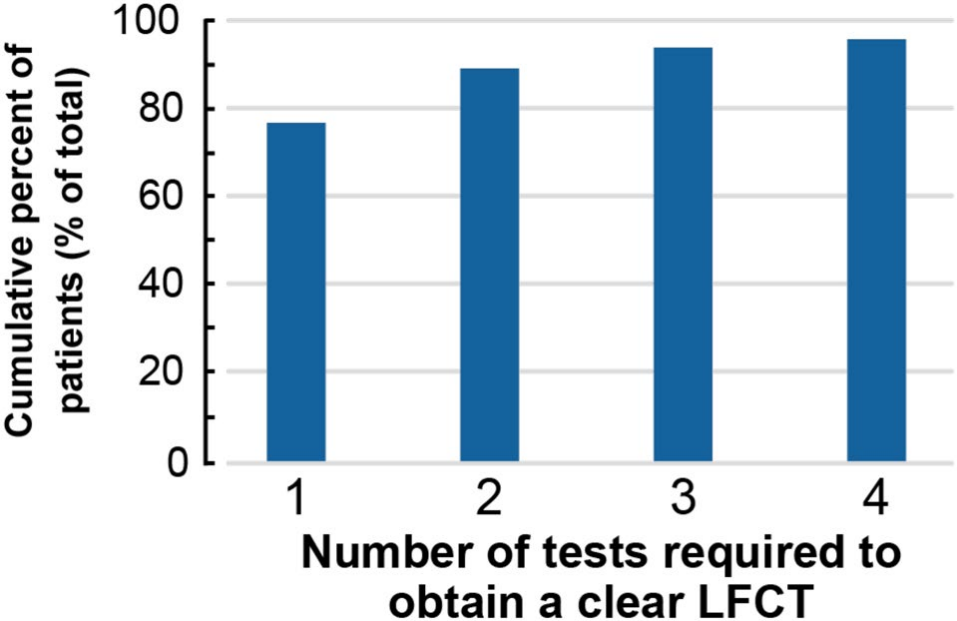
Number of tests required to obtain a clear LFCT. The numbers of tests required to obtain a clear LFCT were shown in Fig. 5. The number of patients in whom we could obtain a clear LFCT value in the first session was 35/46 patients (76.1%), 41/46 patients (89.1%) by the second session, 43/46 patients (93.5%) by the third session, and 44/46 patients (95.7%) by the fourth session. LFCT: lung to finger circulation time.

The number of patients who could be obtained a clear LFCT value in the first session was 35/46 patients (76.1%), 41/46 patients (89.1%) by the second session, 43/46 patients (93.5%) by the third session, and 44/46 patients (95.7%) by the fourth session (Fig. 5).

### Safety

There were no patients who complained of worsening physical condition or pathophysiological disorder. No skin burning was observed.

## Discussion

The main findings of this study were as follows.

First, the intraclass correlation coefficient of our new methods was 0.85, which means that our method has “Excellent reliability.” Second, the measurability of LFCT was as high as 95.7%. Third, LFCT could be obtained in 76.1% of patients at the first session and in 89.1% of patients by the second session. Finally, this procedure could be performed totally safe even in HF patients.

### The intraclass correlation coefficient (ICC) as LFCT reproducibility within the same patient

ICC analysis is commonly used for validation of stable measurement^24–26^. We calculated ICC for the evaluation of intrasubject stability in the measurement of LFCT to show the variability of the obtained LFCT data in a subject is small enough when compared to the inter-subject variability. According to the previous reports, ICC was classified into 3 categories: poor reliability (ICC: < 0.40), fair to good reliability (ICC: 0.40–0.75) and excellent reliability (ICC: > 0.75)^20,27^. Judging from these categorizations, our LFCT measuring method could be classified into “Excellent reliability” range as ICC was 0.85. This means that LFCT values obtained by this method in one patient show high stability and, thus, this method can be considered as highly reliable for measurement of LFCT.

Although our method was classified as having “Excellent reliability”, there remain some points to be improved for practical use. Since we have found that the measured LFCT value deviated out of the expected correlation between LFCT and cardiac index when the fingertip temperature was below 32 °C, it is mandatory to warm a hand in such a condition. As this procedure might be an obstacle for introducing this method into the daily clinical situation, we need to establish some parametric compensation method instead of actual warming. For this purpose, we have to accumulate more data from a big number of patients with various conditions. Since we included outpatients in this study, we couldn’t verify LFCT stability and feasibility over at different timing in a day or on different days. It is critically important to confirm such temporal stability in the future study.

As we had considered that age or cardiac condition might affect the feasibility or stability of LFCT measurement, we separately analyzed our data by dividing the data according to age (≥ 75 years old and < 75 years old), cardiac index by an echocardiogram (≥ 2.2 L/min/M^2^ and < 2.2 L/min/M^2^). With this subgroup analysis, we found that ICC values remained high in any case, implying that this method can be applicable irrespective of the patient condition, though the number of patients was limited. We admit that cardiac output is a highly variable parameter. As we used echocardiogram data from the data in daily clinical setting, we set the time frame of echocardiogram as 4 months. Data of the echocardiogram at the same time point would give us more robust information. We attempted to perform subgroup analysis according to NYHA classification. As there was only one patient in NYHA class 3, and no patient in NYHA class 4, we didn’t perform the NYHA subgroup analysis.

### Secondary evaluation item: feasibility of measurement of LFCT

We could obtain a clear singularity nadir LFCT curve in 76.1% patients at the 1st session and 89.1% of patients by the 2nd session. Combining with the high reproducibility of our method shown by high ICC value, 2- or 3-times repetition of the procedures might be enough to obtain proper LFCT value(s). It would be especially benefitable for frail patients who cannot repeatedly hold their breath for many times. We could obtain at least one measurable LFCT in 44 cases out of 46 cases (measurability was 95.7%). Both of 2 patients were male, in NYHA Class II, preserved cardiac index, and in the 70 s and 80 s. Although the systematic consideration would be difficult for the small number, high age and high sympathetic nervous activity might have affected to the measurability. Especially, considering from our previous study^10^ and the mechanism of measuring LFCT, high sympathetic nerve activity status, which causes peripheral vascular contraction, might have negatively affected to proper measurement of LFCT. We also expect a difficulty in measurement of stable LFCT in patients with atrial fibrillation, because their COs are always varying. Since we didn’t assess the heart rhythm in the current study, we should analyze the effect of arrhythmia on LFCT measurement in the future study.

### The verification with cardiac index

We previously showed that nocturnal LFCT might reflect cardiac index and their changes^13,15^. As the second step, we determined to verify that LFCT measured during daytime using a brief breath hold will correlate with cardiac index. Our previous study^16^ has confirmed that cardiac index and 1/LFCT show a positive correlation tendency in patients with fingertip temperatures above 31 degrees. Combined with the results of studies by other groups^11,12^, we consider that it is highly possible that cardiac index and 1/LFCT are positively correlated. Now that we have confirmed that the reproducibility and safety of our daytime breath holding method are high enough in this presenting study, we would like to conduct research using this method in order to improve the accuracy of the measurement.

### Safety

There were no patients who showed worsening their condition after repeated breath holding measurement even though the patients were senile and with cardiac diseases, which was also shown in our previous study^16^. We strongly believe that LFCT could be safely measured with our method as long as physicians pay minimum attention, like not to hold breath too long or not to select patients who show shortness of breath due to cardiac disease.

### Sample size

The sample size of the present study was determined on the basis of feasibility criteria instead of statistical criteria^28^. Since the purpose of our study wasn't to verify a predetermined hypothesis like whether the ICC is greater than 0.60, we didn't determine the sample size based on statistical power. However, as a result, the confidence interval of ICC and the width was 0.77–0.91 and 0.14 respectively, from which the conclusion of the present study wouldn't be altered. Therefore, we consider that the sample size of this study was sufficient to evaluate the reliability.

### The potential advantages of our method

There are some potential advantages to our method compared with other non-invasive cardiac output (CO) estimations. Easy to handle for general end-users, no required preparation other than the device, and portability are the most appealing feature. Our method can be conducted even at home. Several methods to estimate CO non-invasively exist, like the inert gas rebreathing (IGR)^29^, impedance testing^30^, volume clamp methods^31^. But the IGR method needs to prepare gases like nitrous oxide and devices like an infrared photoacoustic gas analyzer. Impedance methods are affected by various factors other than cardiac output like sensor position, body dimensions, and body posture. And lung congestion and emphysema will affect the impedance method. Using the volume clamp method needs complex procedures for measurement and difficult to handle for general end-users. Pulse wave transit time analysis method and bioimpedance method are also suggested non-invasive CO measurement methods. But pulse wave transit time was reported a poor correlation with CO measured by a standard method in significantly reduced vascular resistance condition^8^. Bioreactance method was also affected by lung injuries and fluid accumulation within the thorax^32^ other than actual CO changes.

### Limitations

We must consider some limitations included in this study. First, the variety of patients’ background cardiac diseases was limited. Especially, there were no HF patients with NYHA Class IV symptom, whose blood flow to finger-tip must be too low to obtain a proper signal. Additional research including this class of patients is necessary. Second, the number of patients was small due to the limitation of time. We need a larger population-based study to obtain various parameters and it would help to establish a parametric compensation model applicable for those whose finger-tip temperature is below 32 °C. Finally, since we analyzed only LFCT data with good quality in this study, ICC might have shown the better results compared to taking all the data without selection.

## Conclusion

LFCT measurement with our method was stable enough to show high ICC and safely feasible in most cardiac patients. This method would be able to apply in an actual clinical setting as a minimum number of repeated procedures to obtain a measurable LFCT was as low as 2 times for 89.1% of patients. With this method of LFCT measurement and further development of practical method for translating this value to CO, treatment of cardiac patients especially with HF would be facilitated.
